# Longitudinal Growth Following Treatment for Osteosarcoma

**DOI:** 10.1080/13577149878073

**Published:** 1998-06

**Authors:** W. Paul Cool, Robert J. Grimer, Simon R. Carter, Roger M. Tillman, A. Mark Davies

**Affiliations:** 1 The Royal Orthopaedic Hospital Oncology Service The Royal Orthopaedic Hospital Bristol Road South Northfield, Birmingham B31 2AP UK; 2 Walnut Cottage Upton Magna Shropshire SY4 4TZ UK

## Abstract

*Purpose.* The purpose of this study was to analyse the height at diagnosis and growth in 72 skeletally immature children who had been treated for osteosarcoma in the area of the knee.

*Subjects.* Of the patients, the average age at diagnosis was 10 years in girls and 12 years in boys. All children received neo-adjuvant chemotherapy, and had limb salvage by endoprosthetic replacement.

*Results and conclusion.* The results of this study indicate that there is no evidence that children with osteosarcoma are taller at diagnosis than their normal counterparts. However, there was a marked retardation in growth in the year following the administration of cytotoxic chemotherapy. There were 19 children who reached skeletal maturity. The final height in these children was not significantly different from the normal population.


*Sarcoma* (1998) **2**, 115–119

ORIGINAL ARTICLE

### Longitudinal growth following treatment for osteosarcoma

W. PAUL COOL, ROBERT J. GRIMER, SIMON R. CARTER, ROGER M. TILLMAN & A. MARK DAVIES

*The Royal Orthopaedic Hospital Oncology Service, The Royal Orthopaedic Hospital, Bristol Road South, Northfield, Birmingham B31 2AP, UK*

**Abstract**

*Purpose.* The purpose of this study was to analyse the height at diagnosis and growth in 72 skeletally immature children who had been treated for osteosarcoma in the area of the knee.
*Subjects.* Of the patients, the average age at diagnosis was 10 years in girls and 12 years in boys. All children received neo-adjuvant chemotherapy, and had limb salvage by endoprosthetic replacement.
*Results and conclusion.* The results of this study indicate that there is no evidence that children with osteosarcoma are taller at diagnosis than their normal counterparts. However, there was a marked retardation in growth in the year following the administration of cytotoxic chemotherapy. There were 19 children who reached skeletal maturity. The final height in these children was not significantly different from the normal population.

**Key words:** *height, longitudinal growth, osteosarcoma, chemotherapy.*

### Introduction

It has been reported that children with osteosarcoma are taller than their normal counterparts.[1] However, this has been contradicted by others.[2,3]

Glasser *et al.*[2] showed that children with malignant primary bone tumours had a marked retardation in growth during the year of cytotoxic chemotherapy. They also suggested that their final height might be affected.

It was the purpose of this study to analyse the height at diagnosis and the growth that had occurred in skeletally immature children with an osteosarcoma around the knee who have been treated by limb salvage at our centre.

### Subjects and methods

All skeletally immature children who were diagnosed between 1981 and 1994 as having an osteosarcoma in the area of the knee, that had been treated at our centre by limb-salvage surgery, were included in this study. Patients were excluded if they died within 1.5 years of diagnosis, or if height measurements were unavailable.

At diagnosis, all patients were fully staged and had their bone age estimated according to the Greulich and Pyle[4] method. Height was also measured in the patients, while standing with a level pelvis.[5]

Following diagnosis, all patients received neo-adjuvant chemotherapy according to the then current protocol. This was normally cisplatinum and doxorubicin or cisplatinum, doxorubicin and high-dose methotrexate.[6]

After three cycles of chemotherapy (usually 9 weeks following diagnosis), patients were restaged and underwent limb-salvage surgery. Limb salvage was performed by resection of the tumour and endoprosthetic replacement. The expected growth in the resected segment was calculated with the aid of the bone age and data provided by Tupman.[7] If the expected growth in the resected segment exceeded 3 cm, an extensible endoprosthetic replacement was inserted,[8–10] otherwise a non-extensible replacement that allowed some normal growth to continue was used.[10–12]

Patients were discharged after 2 weeks and had a further three cycles of chemotherapy. All children were followed-up in the outpatient clinic and had their height estimated at regular intervals until they reached skeletal maturity. Children who had an extensible prosthesis had regular lengthenings in order to maintain limb length equality. On average, two lengthening operations per year, from time of

Correspondence to: W. P. Cool, Walnut Cottage, Upton Magna, Shropshire SY4 4TZ, UK. Tel: +44 1691 404000 page 147 (daytime) +44 1743 709210; E-Mail: pcool@netcomuk.co.uk.

1357-714X/98/020115-05 ©1998 Carfax Publishing Ltd diagnosis until they reached skeletal maturity, were performed.[13]

The heights at diagnosis of all children were plotted in the growth charts for British children[14,15] according to guidelines given by Cole.[16]

The children were split into three groups. One group of patients below the 25th percentile, one group between the 25th and 75th percentiles and one group above the 75th percentile. Statistical analysis was performed with the aid of the Chi-square test.

The heights of the children 1 and 5 years following diagnosis, and at skeletal maturity, were also plotted in the growth charts. Statistical analysis was performed in a manner identical to that described above.

The standard deviation score (or $Z$ score) is the actual height of the patient minus the mean height of the population for that chronological age divided by the appropriate standard deviation. Therefore, a standard deviation score of 0 indicates that the patient has an average height for age and gender. Similarly, a child with a score of $+1$, is one standard deviation above average for age and gender. The difference in standard deviation score at diagnosis and 1 year following treatment is a measure for the velocity of growth. A difference in standard deviation score of 0 indicates that the child had an average velocity of growth for that age range.

The standard deviation score was calculated for all patients who had their height estimated at diagnosis and after 1 year following treatment. The difference in standard deviation score after 1 year was calculated (negative is a decrease in standard deviation score). Similarly, the difference in standard deviation score was calculated 5 years following treatment and at skeletal maturity.

All patients had their bone age estimated at time of diagnosis. The bone age was estimated according to the method described by Greulich and Pyle[4] and compared to chronological age.

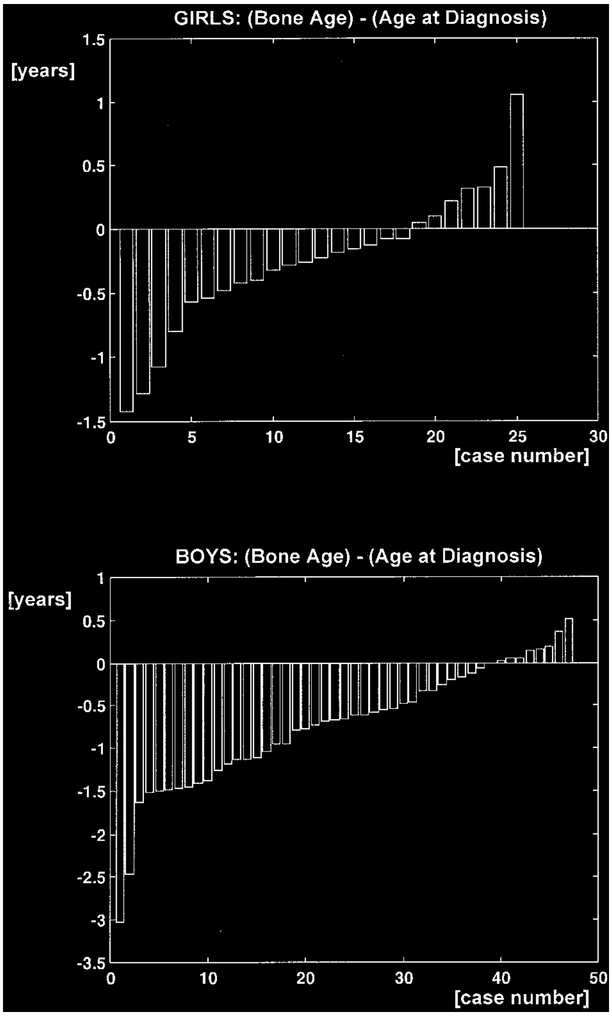

**Fig. 1.** *Difference in bone age and chronological age at diagnosis in 72 children with an osteosarcoma in the area of the knee. A negative value indicates that the bone age was less than the chronological age.*

### Results

Seventy-two children were identified from the medical records. There were 47 boys and 25 girls. The average age at diagnosis was 12.1 years (range 5.8–15.3) in boys and 9.9 years (range 5.8–13.5) in girls. In 35 cases the left leg was affected and in 37 cases the right leg. There were 45 children who had a distal femoral, and 27 who had a proximal tibial, osteosarcoma. Limb salvage was by an endoprosthetic replacement that could be lengthened in 50 children. The remaining 22 children had an endoprosthetic replacement that could not be lengthened, but that allowed for some growth to continue.[10–12]

All children received neo-adjuvant chemotherapy. Forty-three children received cisplatinum and doxorubicin only, and 17 children received cisplatinum and doxorubicin in combination with high-dose methotrexate.[6] Of the remaining 12 children, nine had chemotherapy according to the regime proposed by Rosen *et al.*[17] and three children had alternative treatment protocols.

There were 22 children who eventually died. Twenty deaths were due to metastatic disease, one due to doxorubicin-induced cardiomyopathy, and one patient died due to extensive ileofemoral thrombosis. All but one patient died within 5 years of diagnosis, but no patient died prior to 1.5 years following diagnosis.

The bone age at diagnosis was on average 9.7 years (range 6–13) in girls and 11.3 years (range 5–14) in boys. As can be seen in Fig. 1, the vast majority of children had a bone age that was less than the chronological age (on average 0.25 years in girls and 0.73 years in boys). This difference was statistically significant (sign test, $p < 0.05$).

There were 63 patients who had their height estimated at diagnosis. The results are shown in Fig. 2. There was no statistically significant difference

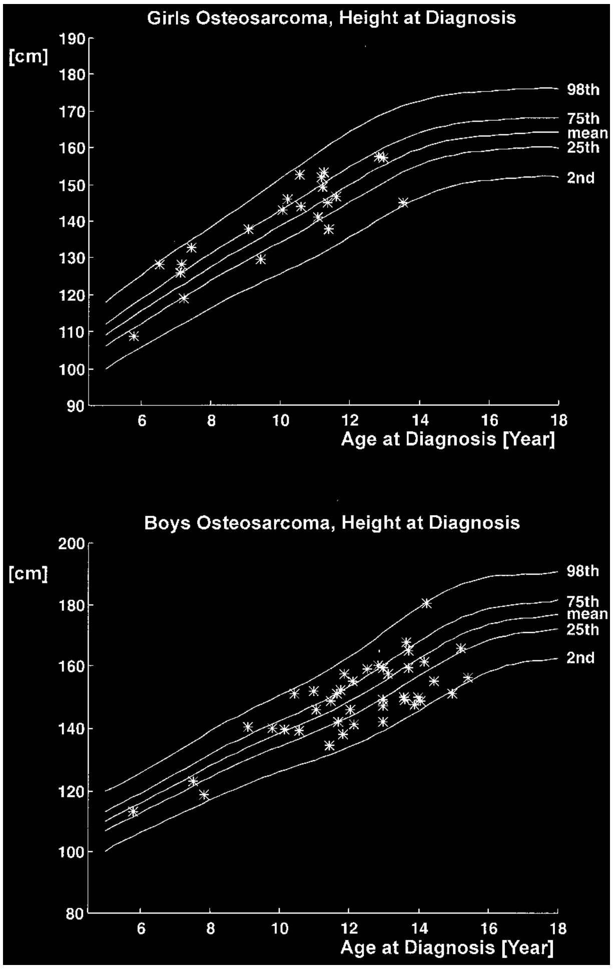

**Fig. 2.** *Height at diagnosis in 63 children with an osteosarcoma in the area of the knee plotted in the growth charts for the normal population.*[14,15]

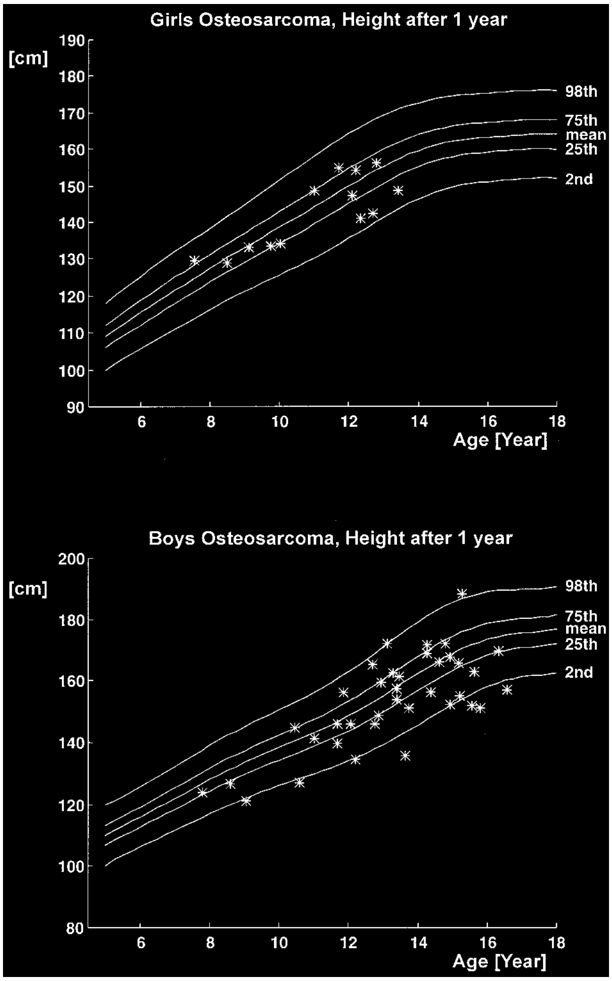

**Fig. 3.** *Height 1 year following diagnosis in 50 children who received neo-adjuvant chemotherapy for an osteosarcoma in the area of the knee plotted in the growth charts for the normal population.*[14,15]

between the children in our study and the population norm.[14,15]

In 50 children, the height was measured 1 year following diagnosis (Fig 3). The heights in these children were also not significantly different from the normal population.[14,15] Forty-one of these 50 children also had their height estimated at diagnosis. As can be seen in Fig. 4, the vast majority of children had a standard deviation score that was less than the standard deviation score at diagnosis. In girls, the standard deviation score 1 year following diagnosis was, on average, 0.67 less than that at diagnosis, whilst this was 0.30 less in boys. This difference was statistically significant (sign test, $p < 0.05$), indicating that chemotherapy causes retardation in growth in the year following treatment.

At 5 years following diagnosis, 18 children had their height measured. The heights in these children were also not significantly different from the normal population.[14,15] Fifteen of these children also had their height measured at diagnosis. In girls, the standard deviation score was, on average, 0.17 less than the standard deviation score at diagnosis. Boys had a standard deviation score that was on average 0.25 less than that at diagnosis.

There were 19 children in our study who reached skeletal maturity. The final height in these children was not significantly different from the normal population (Fig 5). Sixteen of these children also had their height estimated at diagnosis. The standard deviation score in girls who reached skeletal maturity was, on average, 0.48 higher than that at diagnosis, whilst this was 0.24 higher in boys.

These results indicate that the surviving children subsequently make up for the retardation in growth caused by the chemotherapeutic treatment. Although the number of children who reached skeletal maturity is relatively small, final height does not seem to be different from the population norm.[14,15]

### Discussion

This study failed to reproduce the earlier findings of Fraumeni,[1] suggesting that children with osteosar-

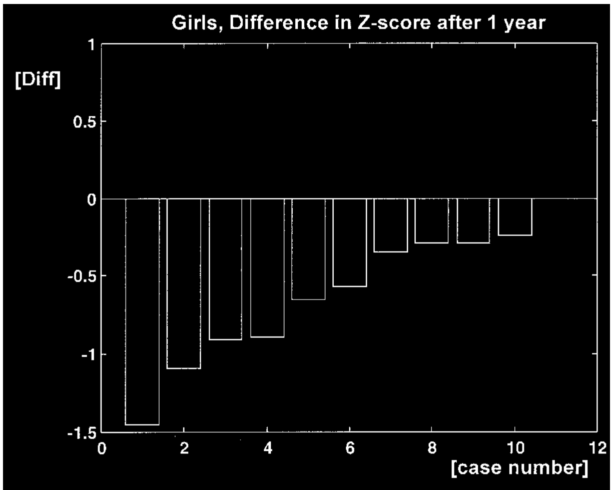

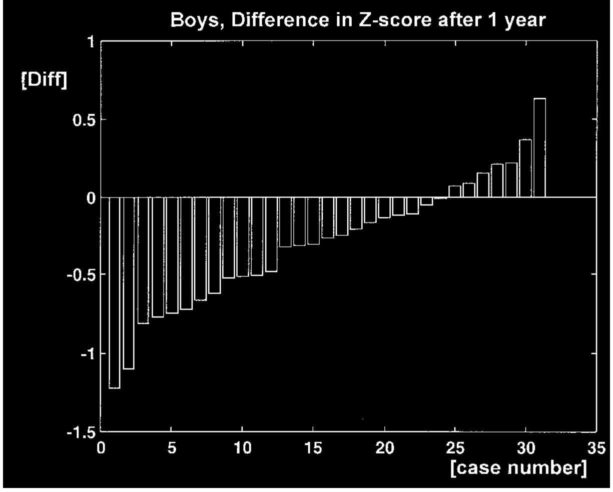

**Fig. 4.** *Difference in standard deviation score after 1 year in 41 children who had their height estimated at diagnosis and after 1 year. A negative value indicates that the child had a less than average growth for age and gender.*

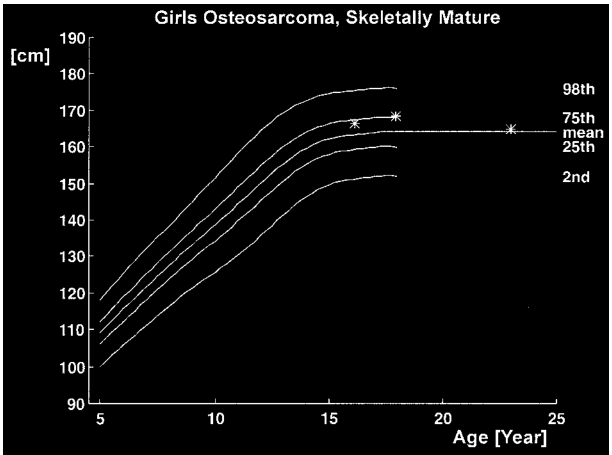

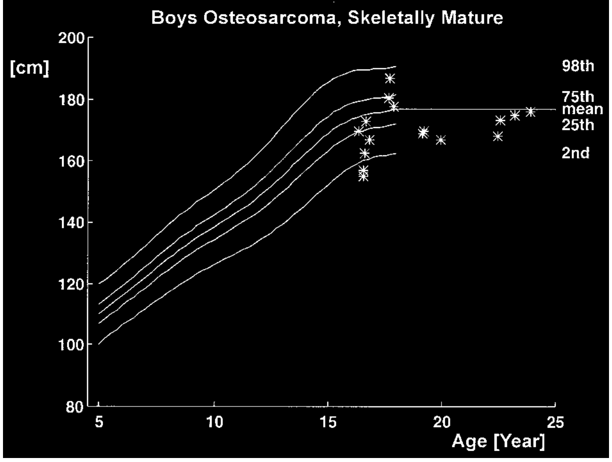

**Fig. 5.** *Height at skeletal maturity in 19 children who have been treated for an osteosarcoma in the area of the knee plotted in the growth charts for the normal population.*[14,15]

coma are taller than the normal population. Others[2,3] also could not find such a relation.

Osteogenic sarcoma is more common in the second decade of life, during increased skeletal growth. It seems therefore likely that the incidence of osteosarcoma is related to the velocity of growth at time of diagnosis rather than the height at diagnosis. Unfortunately, no pre-diagnostic height measurements were available from the children in this study. This makes it difficult to draw any conclusions. However, it is interesting to note that the vast majority of children had a bone age at diagnosis that was less than their chronological age. It might be that these children were catching up on their relative skeletal immaturity and had an increased velocity of growth at time of diagnosis.

The height 1 year following chemotherapy treatment was also not significantly different from the population norm.[14,15] However, there was a significant reduction in standard deviation score, indicating that growth is retarded following the administration of cytotoxic chemotherapy. This further illustrates that actual height is a poor indicator of growth retardation and that the velocity of growth is a more sensitive measure.

The final height of the children in this study was similar to the normal population.[14,15] Furthermore, the difference in standard deviation score was, on average, positive. This indicates that these children subsequently make up for the retardation in growth caused by the chemotherapy treatment.

In conclusion, there is no evidence that children with osteosarcoma are taller than their normal counterparts. Growth is retarded in the year following chemotherapy treatment, but the final height in these children is not significantly different from the normal population.

### References

1 Fraumeni JF. Stature and malignant tumors of bone in childhood and adolescence. *Cancer* 1967; 20:967–73.
2 Glasser DB, Duane K, Lane JM, *et al.* The effect of chemotherapy on growth in the skeletally immature individual. *Clin Orthop* 1991; 262:93–100.
3 Operskalski EA, Preston-Martin S, Henderson BE, *et*

*al.* A case-control study of osteosarcoma in young persons. *Am J Epidemiol* 1987; 126:118–26.
4 Greulich WW, Pyle SI. *Radiographic atlas of skeletal development of the hand and wrist*. 2nd ed. Stanford: Stanford University Press, 1966.
5 Burwell RG, Vernon CL, Dangerfield PH. Skeletal measurement. In: Owen R, Goodfellow J, Bullough P, eds. *Scientific foundations of orthopaedics and traumatology*. London: William Heinemann, 1980: 317–29.
6 Bramwell VHC, Burgers M, Sneath R, *et al.* A comparison of two short intensive adjuvant chemotherapy regimens in operable osteosarcoma of limbs in children and young adults: The first study of the european osteosarcoma intergroup. *J Clin Oncol* 1992; 10:1579–91.
7 Tupman GS. A study of bone growth in normal children and its relationship to skeletal maturation. *J Bone Joint Surg (Br)* 1962; 44B:42–67.
8 Scales JT, Sneath RS. The extending prosthesis. In: Coombs R, Friedlandler G eds. *Bone tumour management*. London: Butterworths, 1987: 168–77.
9 Scales JT, Sneath RS, Wright KWJ. Design and clinical use of extending prostheses. In: Enneking WF, ed. *Limb salvage in musculoskeletal oncology*. New York: Churchill Livingstone, 1987: 52–61.
10 Sneath RS, Carter SR, Grimer RJ. Growing endoprosthetic replacements for malignant tumours. In: Langlais F, Tomeno B, eds. *Limb salvage—major reconstructions in oncologic and nontumoral conditions*. Berlin: Springer-Verlag, 1991: 573–8.
11 Cool WP, Grimer RJ, Carter SR, *et al.* The sliding component. *J Bone Joint Surg (Br)* 1995; 77B (Suppl 3):330.
12 Inglis AE, Walker PS, Sneath RS, *et al.* Uncemented intramedullary fixation of implants using polyethylene sleeves, a roentgenographic study. *Clin Orthop* 1992; 284:208–14.
13 Cool WP, Grimer RJ, Carter SR, *et al.* The outcome of extensible endoprosthetic replacements of the distal femur and proximal tibia. *J Bone Joint Surg (Br)* 1996; 78B (Suppl 1):58.
14 Child Growth Foundation. Boys growth chart, United Kingdom cross sectional reference data 1994/1.
15 Child Growth Foundation. Girls growth chart, United Kingdom cross sectional reference data 1994/1.
16 Cole TJ. Do growth centile charts need a face lift? *Br Med J* 1994; 308:641–2.
17 Rosen G, Caparros B, Huvos AG, *et al.* Preoperative chemotherapy for osteogenic sarcoma: selection of postoperative adjuvant chemotherapy based on the response of the primary tumor to preoperative chemotherapy. *Cancer* 1982; 49:1221–30.
